# A Novel Biosensor for Evaluation of Apoptotic or Necrotic Effects of Nitrogen Dioxide during Acute Pancreatitis in Rat

**DOI:** 10.3390/s100100280

**Published:** 2009-12-30

**Authors:** Dagmara Jacewicz, Aleksandra Dabrowska, Dariusz Wyrzykowski, Joanna Pranczk, Michal Wozniak, Jolanta Kubasik-Juraniec, Narcyz Knap, Kamila Siedlecka, Alexander J. Neuwelt, Lech Chmurzynski

**Affiliations:** 1 Department of General and Inorganic Chemistry, University of Gdańsk, Sobieskiego 18/19, 80-952 Gdańsk, Poland; E-Mails: alex@chem.univ.gda.pl (A.D.); daro@chem.univ.gda.pl (D.W.); jpranczk@interia.pl (J.P); lech@chem.univ.gda.pl (L.Ch.); 2 Department of Medical Chemistry, Medical University of Gdańsk, Dębinki 1, 80-211, Gdańsk, Poland; E-Mails: mwozniak@amg.gda.pl (M.W.); narcyz@amg.gda.pl (N.K.); 3 Department of Electron Microscopy, Medical University of Gdańsk, Dębinki 1, 80-211, Gdańsk, Poland; E-Mail: kubasik@amg.gda.pl; 4 Department of Histology & Immunology, Medical University of Gdańsk, Dębinki 1, 80-211, Gdańsk, Poland; E-Mail: ksiedlecka@amg.gda.pl; 5 Department of Neurology, Oregon Health & Sciences University, Portland OR 97239, USA; E-Mail: aneuwelt@gmail.com

**Keywords:** acute pancreatitis, nitrogen dioxide, l-arginine, Cr(III), rats, levels of inflammatory mediators, evaluation of apoptotic or necrotic effects

## Abstract

The direct and accurate estimation of nitric dioxide levels is an extremely laborious and technically demanding procedure in the molecular diagnostics of inflammatory processes. The aim of this work is to demonstrate that a stop-flow technique utilizing a specific spectroscopic biosensor can be used for detection of nanomolar quantities of NO_2_ in biological milieu. The use of novel compound *cis*-[Cr(C_2_O_4_)(AaraNH_2_)(OH_2_)_2_]^+^ increases NO_2_ estimation accuracy by slowing down the rate of NO_2_ uptake. In this study, an animal model of pancreatitis, where nitrosative stress is induced by either 3g/kg bw or 1.5 g/kg bw dose of l-arginine, was used. Biochemical parameters and morphological characteristics of acute pancreatitis were monitored, specifically assessing pancreatic acinar cell death mode, NO_2_ generation and cellular glutathione level. The severity of the process correlated positively with NO_2_ levels in pancreatic acinar cell cytosol samples, and negatively with cellular glutathione levels.

## 1. Introduction

Acute pancreatitis (AP) is a common and potentially fatal disease consisting of diffuse inflammatory edema of the pancreas [1]. Acinar cell degeneration during AP, from the morphological point of view, may involve apoptosis (edematous form of AP) or necrosis (necrotic form of AP) [2].

The chemistry and pathophysiology underlying the oxidative stresses that contribute to either apoptotic or necrotic cell death in AP are not well characterized. Levels of inflammatory mediators, namely interleukin 1 (IL-1), IL-6 and tumor necrotic factor α (TNF α), begin to elevate in patients’ serum within one hour of the onset of AP [3]. Interleukin-6 (IL-6) concentration in patients with AP correlates with the severity of the disease. These inflammatory mediators have been found to trigger the induction of inducible nitric oxide synthase (iNOS) resulting in the overproduction of NO [4].

Some evidence indicates that reactive nitrogen species (RNS) may act as important signal transducers in the induction of apoptosis in acinar cells. Mizunuma *et al.* [5] were the first to report that single intraperitoneal (ip) administration of l-arginine (l-arg) resulted in the selective injury of pancreatic acinar cells while leaving beta cells intact. Moreover, the formation of nitrogen dioxide radical in the pancreas has been pivotally implicated in the course of necrotic AP induced by l-arg [1].

A human pulmonary type II-like epithelial cell line A-549 has been found to acquire considerable resistance to exogenous NO_2_, perhaps due to considerably higher levels of cellular glutathione [6]. On the contrary, HUVEC and C-21 cell lines display both low pre-exposure GSH (reduced glutathione) levels and a high sensitivity to NO_2_ [6]. However, the reasons for intracellular variation in NO_2_ sensitivity remain poorly understood. Interestingly, the effect of endogenously generated nitrogen dioxide on intracellular glutathione levels has not been studied.

Little is known about the generation of RNS in AP. An increased expression of iNOS during the early onset of AP is observed mainly within vascular muscle cells [4]. Also, nitrotyrosine being a hallmark of NO_2_ generation was detected in the perivascular region during l-arginine induced AP, which seems to be a good model for the study of nitrosative stress in the course of the disease [7].

Acute pancreatitis develops first locally, within perivascular regions of the pancreas, and only later generates a systemic response. Assessing the severity of acute pancreatitis is an important initial step in the management of AP patients.

There is currently no reliable marker of AP severity. The most commonly used animal model of edematous AP is induced using a secretagogue cerulein. Induction of necrotic AP is commonly performed via the infusion of taurodeoxycholate into the biliary and pancreatic duct. It is believed that in severe AP, premature activation of intra-acinar zymogens leads to pancreatic necrosis.

Because of the involvement of RNS and ROS (reactive oxygen species) in both the edematous and necrotic form of AP, these molecules are being examined as potential markers for disease severity. It is thought that iNOS is involved in early inflammatory cytokine induction, an effect observed in animal models as well as in affected patients. There is also growing evidence that patients with AP have increased NO generation.

To inquire whether NO_2_, a free radical thought to primarily react with reduced glutathione (GSH) in the cell, can modulate an apoptotic/necrotic switch in pancreatic acinar cells, Wistar rats were exposed to different concentrations of l-arginine. High (3 g/kg bw) and low (1.5 g/kg bw) doses of l-arginine were chosen to respectively generate higher or lower levels of NO_2_ via the iNOS pathway in order to deplete GSH being a principal cellular antioxidant, and thus induce either necrotic or apoptotic cell morphology. Consequently, the chemical and physiologic characteristics of the treated rats were evaluated, primarily focusing on the role of RNS in apoptotic or necrotic events in the studied animals. Moreover, the efficacy of 4-OH TEMPO in reacting and scavenging NO_2_ radicals was studied.

In our previous studies we have established an ultrasensitive micromethod of ^•^NO_2_ detection based on the interaction of this radical with a coordination compounds of Cr(III) with biologically active organic ligands [8]. In view of different duration of the *in vivo* reactions, the stopped-flow technique was employed for the detection of ^•^NO_2_. The primary purpose of these studies was to determine the kinetics and mechanism of ^•^NO_2_ uptake by co-ordination compounds of general formula *cis*-[Cr(C_2_O_4_)(L-L)(OH_2_)_2_]^+^, where L-L denotes aminodeoxyglycopyranoside derivatives. The kinetic measurements of the nitrogen dioxide radical uptake carried out by using the stopped-flow technique for a variety of biological ligands allowed to establish the mechanism of substitution reactions in particular coordination compounds in aqueous solution, thus providing assessment whether or not the systems under study can constitute model mimics of enzymatic reactions occurring in biological systems.

Detection of ^•^NO_2_ generated in cytosols isolated from the pancreas of the rat intoxicated with l-arg was successful, however, ^•^NO has been found not to interact with two anomers of methyl 3-amino-2,3-dideoxyhexopyranoside, *i.e.*, with α-d-arabino (AaraNH_2_) [9] and β-d-arabino (BaraNH_2_) configurations [10–12]. While carrying out the measurements of ^•^NO_2_ uptake by the two complex ions *cis*-[Cr(C_2_O_4_)(AaraNH_2_)(OH_2_)_2_]^+^ or *cis*-[Cr(C_2_O_4_)(BaraNH_2_)(OH_2_)_2_]^+^ it was noted that for all studied samples the approximated curve decayed biexponentially. It should be stressed that the studied reaction proceeded in two steps. The observable rate constants, for the first (k_1obs_) and second steps (k_2obs_), were obtained by fitting the rate data to the same consecutive reaction model. Global value analysis of the observable rate constants for both steps was based on the model of consecutive reactions of type: A →B →□C. In the first step, an intermediate compound B was formed and subsequently converted to a final product C, characteristic for the second step. The same reaction pathway in the biological sample was confirmed by the spectral and kinetic analysis for both complexes. Nitrogen dioxide levels in the cytosolic fraction of pancreatic acinar cells were determined on the basis of a standard curve created according to the method of Jacewicz *et al.* [12].

## 2. Results and Disscusion

### 2.1. Kinetics of ^•^NO_2_ Uptake Generated from Pancreatic Cytosol Fractions

The presented study is a continuation of our kinetic studies on the mechanisms of gas uptake reactions by Cr(III) co-ordination compounds with bioactive ligands [8]. The selection of chromium(III) as the center of co-ordination permits the creation of inert complexes that undergo relatively slow transformations at ambient temperature, thus facilitating investigations on the kinetics and mechanism of the studied processes. The synthesis of anomeric methyl 3-amino-2,3-dideoxy-α-d-*arabino-*hexopyranoside co-coordinated to Cr(III) turned out to be successful for the detection of *^•^*NO_2_ generated in the cytosol isolated from the pancreas of rats intoxicated with L-arg. Importantly, *^•^*NO has been found not to interact with this ligand. While carrying out measurements of *^•^*NO_2_ uptake by cis-[Cr(C_2_O_4_)(AaraNH_2_)(OH_2_)_2_]^+^, it was noted that for all studied cytosol samples the approximated curve decayed bi-exponentially. It should be stressed that the studied reaction proceeded in two steps. The observable rate constants, k_1obs_ for step 1 and k_2obs_ for step 2, were obtained by fitting the rate data to the consecutive reaction model. Global value analysis of the observable rate constants for both steps was based on the model of consecutive reactions of the type:

$$A\overset{k_{1}}{\to }B\overset{k_{2}}{\to }C$$

**Figure 1.**
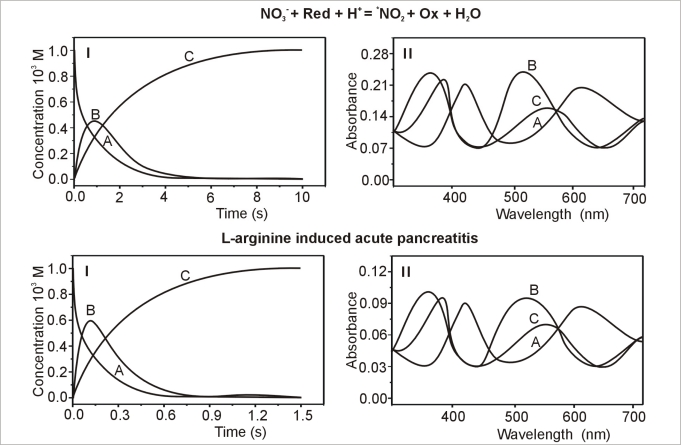
(I) Comparison of kinetic and spectral characteristics of reactants in simple inorganic *^•^*NO_2_ generating system and pancreatic cytosol fraction. (II) Absorption spectra of the reactants A, B and C at pH = 7.12, [NO_2_] = 2.48E-5, T = 20 °C.

In the first step, an intermediate compound B was formed and was subsequently converted to a final product C, characteristic of the second step. The results of the global analysis, for *^•^*NO_2_ uptake by *cis-*[Cr(C_2_O_4_)(AaraNH_2_)(OH_2_)_2_]^+^ within the consecutive reaction model are presented in Figure 1. These data support the proposed reaction mechanisms. This reaction pathway was also confirmed by spectral and kinetic analysis of the ion complex (Figure 1).

The determined acidity constants for *cis-*[Cr(C_2_O_4_)(AaraNH_2_)(OH_2_)_2_]^+^ are as follows: K_1_= 2.75E-7 ± 3.56E-9, and K_2_ = 2.82E-9 ± 7.45E-11. Taking into account the observable rate constants, a mathematical model for *^•^*NO_2_ uptake reaction and the molecular mechanism of the detection of *^•^*NO_2_ were designed [12].

### 2.2. Biological Study

**Figure 2.**
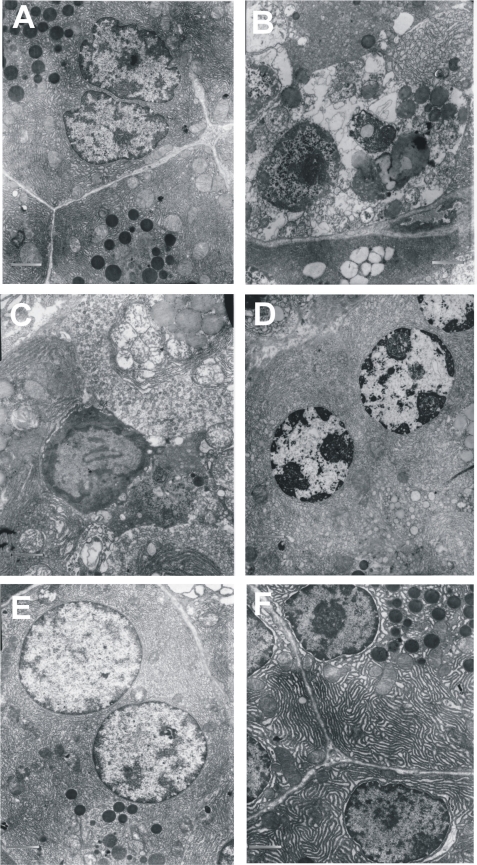
Morphological changes of the pancreatic acinar cells upon l-arginine induced necrosis and apoptosis. Protective effect of 4-OH-TEMPO.

Distinct differences between control and l-arginine exposed pancreatic acinar cells were found in the structure and localization of cytoplasmic organelles. Control cells displayed abundant undilated sheets of rough endoplasmic reticulum. They secreted numerous electron dense zymogen granules (2G) from the apical pole of acinar cells (Figure 2A). Small granular or elongated filamentous mitochondria with well preserved cristae, concentrating around zymogen granules, were also visible (Figure 2A). Specimens of pancreatic tissue taken 24 h after exposure to 3 g or 1.5 g of l-arg per 1 kg bw, demonstrated markedly different morphology. Namely, 3 g of l-arg resulted in the appearance of cells with loss of plasma membrane integrity and occasional cleavage of organelles into interstitium - a necrotic hallmark (Figure 2B). Further, these cells also had dilatated endoplasmic reticulum and Golgi complexes, degraded zymogen granules, and nuclear membrane lysis resulting in chromatin being assembled below the nuclear envelope (Figure 2B). Furthermore, pancreatic acinar tissue infiltration by macrophages, disintegrated endoplasmic reticulum, and clusters of mitochondria with peripherally visible remnants of cristae were also seen in cells treated with 3 g/kg l-arg and suggest the presence of inflammatory processes affecting necrotic acinar cells (Figure 2C). On the other hand, cells exposed to 1.5g of l-arg /kg bw displayed processes more consistent with apoptotic cell death, including nuclei with peripheral crescents of compact chromatin, as well as cytoplasm that appeared condensed (Figure 2D).

4-OH-TEMPO has been used as a non-peptide mimic of SOD [13] in order to prevent inflammatory superoxide reactivity. Bonini *et al.* speculate that 4-OH-TEMPO will be unable to react with NO_2_ [14]. On the other hand, Goldstein *et al.* [15] demonstrated the efficacy of 4-OH-TEMPO in scavenging NO_2_ and not O_2_^•^^−^ under physiological conditions.

It was observed that 4-OH-TEMPO had a highly protective effect on the ultrastructure of acinar cells in AP upon high (3 g/kg, Figure 2E) or low (1.5 g/kg, Figure 2F) dose l-arginine.

GSH is the main intracellular scavenging compound of oxidative chemicals, and thus is indirectly responsible for lactate dehydrogenase release from different cell lines exposed to NO_2_, a well accepted biomarker of cell necrosis [6]. However, several studies also demonstrate a protective effect of GSH in preventing apoptotic response of oxidatively challenged cells.

In order to elucidate the role of GSH and NO_2_ in edematous and necrotic acute pancreatitis, Wistar rats were treated with l-arginine at a dose of 1.5 or 3 g/kg bw respectively. After an induction period, samples of pancreata were examined for intracellular GSH and NO_2_ concentrations, and changes of these parameters were studied with respect to observed modes of cell death.

**Figure 3.**
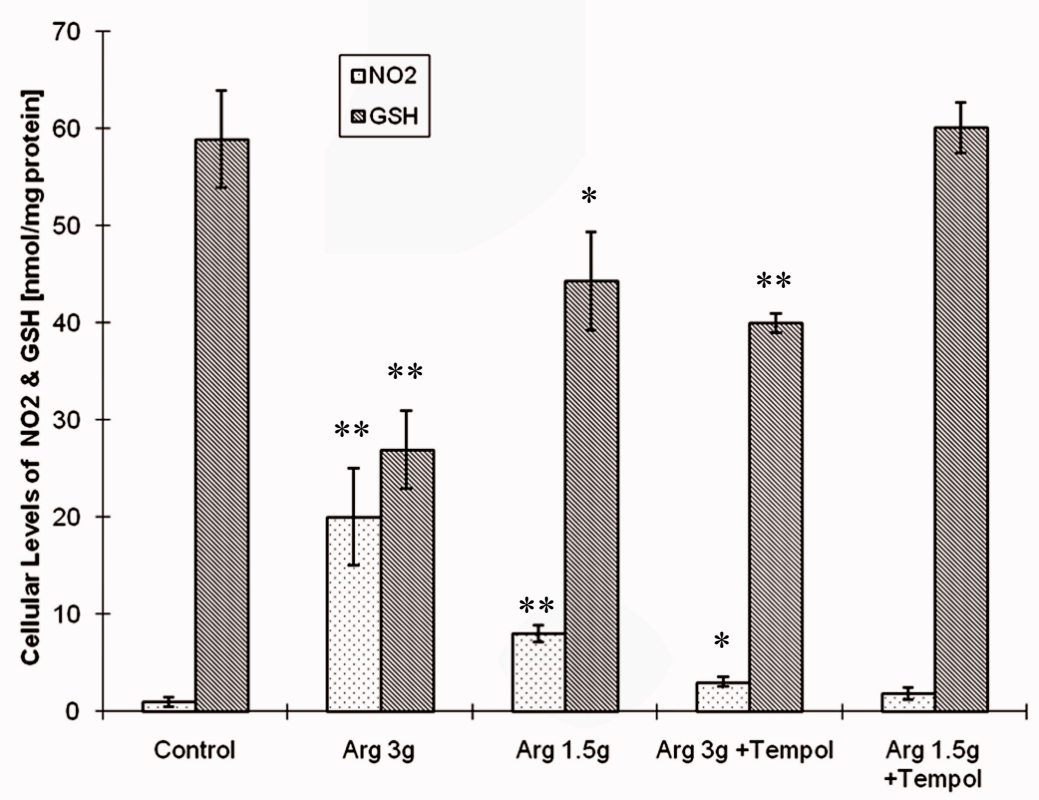
NO_2_ and GSH levels in pancreatic cytosol from rats administered 3 or 1.5 g/kg bw of l-arginine. Protective effect of 4-OH-TEMPO (Tempol). Statistical significance between treatment and respective control group was indicated by an asterisk: *P < 0.05 or **P < 0.01.

Nitrogen dioxide levels in the cytosolic fraction of pancreatic acinar cells were determined on the basis of a standard curve created according to the method of Jacewicz *et al.* [8]. Administration of lower and higher doses of l-arg resulted in remarkable increases of NO_2_ concentration, with final concentrations of 6.71 nmol/mg protein and 20 nmol/mg protein, respectively (Figure 3). Exposure to 3 g of l-arg caused a more pronounced depletion of cellular GSH as compared to the lower dose (Figure 3). Tempol proved to effectively protect the cells against NO_2_ generation and consequent GSH depletion.

**Figure 4.**
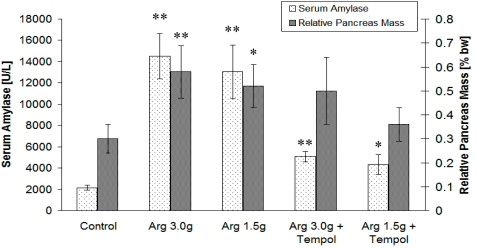
Serum amylase levels and relative pancreatic mass (expressed as percentage of total body weight [%bw]) in l-arginine challenged rats. Protective effect of 4-OH-TEMPO (Tempol). Statistical significance between treatment and respective control group was indicated by an asterisk: *P < 0.05 or **P < 0.01.

A gradual decrease of GSH can be considered a key result of NO_2_ oxidative chemistry, and seems to correspond to morphological determinants of cellular death mode. Moreover, NO_2_ generation and consequent GSH depletion correlated with the clinical severity of AP as assessed by serum amylase activity as well as panreatic edema index, *i.e.*, pancreas mass expressed as percentage of total body weight (Figure 4).

### 2.3. Discussion

An oxidizing cellular microenvironment can cause apoptotic or necrotic cell death. Apoptosis may occur with relatively moderate oxidative stimuli, while necrosis can result from more severe oxidative challenges leading to the loss of the cell’s ability to effectively defend itself against oxidative stresses. Reactive oxygen/nitrogen species generated within an inflamed organ have an unfavorable impact on cells, and may potentially induce apoptosis or necrosis. Because NO_2_, a lipophilic molecule, originates inside pancreatic acinar cells, we postulate that interaction of NO_2_ with highly electrophilic molecules inside the cell, like GSH, is an early pro-necrotic as well as pro-apoptotic event in AP.

Hepatic centrilobular necrosis has been found to correlate with the nitration of a selected group of proteins. Nitration at high levels has been implicated in necrosis while low levels are believed to cause apoptotic cell injury. Direct real-time evaluation of nitration of a green fluorescent protein in solution as well as within cells reveals that heme-peroxidase catalyzed formation of NO_2_ may play a critical role in inflammatory disease. This finding questions the significance of nitration mediated by peroxynitrite [16].

Because intracellular GSH concentrations are far higher than those of all other cellular antioxidants, GSH is considered to be the major cellular defense mechanism against oxidative stress [17]. Recently it was suggested that GSH redox status may be one of the final determinants for the execution of apoptosis [18]. GSH reduction potential has been postulated to be a sort of cellular switch for proliferation, differentiation, apoptosis and necrosis. Using a transmission electron microscope, we observed ultrastructural changes in cellular morphology upon exposure of acinar cells to L-arginine. Among the changes seen were mitochondrial damage, dilatation of endoplasmic reticulum cisternae, and clumping of nuclear chromatin. 4-OH-TEMPO administration prevented these nuclear, endoplasmic reticulum, and mitochondrial changes. Pancreatic acinar cell damage was found to be initiated by significant increases in cytosolic NO_2_ levels resulting from cytosolic GSH depletion. This effect was significantly reduced by the well known radical scavenger 4-OH-TEMPO.

Our results point to a high reactivity of tempol aminoxyl with NO_2_ radicals. Further, our findings suggest that the oxidative chemistry of NO_2_ plays a role in the metabolic switch from apoptotic into necrotic death mode, an effect mediated by depletion of cellular GSH level. 4-OH-TEMPO is effective in limiting acinar cell damage by efficient scavenging of reactive NO_2_ radicals.

Our findings are unique in establishing a sensitive and selective spectroscopic method of qualitative and quantitative NO_2_ estimation in the presence of other reactive nitrogen species. The presented results suggest the need for additional prospective studies of inflammatory diseases in order to establish the universality of NO_2_ contribution to the cellular chemistry and molecular biology of inflammation. Moreover, in the absence of effective anti-NO_2_ directed treatment, studies focusing on the development of therapies that could modify the inflammatory response to NO_2_ would be a major advance.

## 3. Materials and Methods

### 3.1. Reagents

Methyl 3-amino-2,3-dideoxy-α-d-*arabino*-hexopyranoside (AaraNH_2_), and *cis*-[Cr(C_2_O_4_) (AaraNH_2_)(OH_2_)_2_]^+^ were synthesized according to the procedures described in [9,19], respectively. The final uptake product, *cis*-[Cr(C_2_O_4_)(L-L)(ONO_2_)]^−^, was characterized according to a procedure described previously [19].

### 3.2. Animals-Experimental Groups

The experiments performed were approved by the Animal Care Ethical Committee of Medical University of Gdansk. Male Wistar rats weighing 250 g were used. The animals were kept at a constant temperature of 25 °C with a 12-hour light–dark cycle, and were allowed free access to water and standard laboratory chow. The rats were fasted for 16 hrs before injections; access to water was kept until euthanasia. The animals were randomly divided into five experimental groups:

1. control rats that received 0.9% NaCl *(ip)* (n = 8),
2. rats injected *(ip)* with L-arg/HCl solution (pH = 7.4) at a dose of 1.5 g of free amino acid per 1 kg bw (n = 8),
3. rats injected *(ip)* with L-arg/HCl solution (pH = 7.4) at a dose of 3.0 g of free amino acid per 1 kg bw (n = 8),
4. rats injected *(ip)* with 20 μg 4-OH-TEMPO dissolved in 0.9% NaCl; 20 min. later the rats were injected *(ip)* with L-arg/HCl solution (pH = 7.4) at a dose of 1.5 g of free amino acid per 1 kg bw (n = 8),
5. rats injected *(ip)* with 20μg 4-OH-TEMPO dissolved in 0.9% NaCl; 20 min. later the rats were injected *(ip)* with L-arg/HCl solution (pH = 7.4) at a dose of 3.0 g of free amino acid per 1 kg of bw (n = 8),

24 hrs after the injection, the rats were anesthetized, blood samples were collected from the heart, and their pancreas excised. The rats were then killed by exsanguination.

#### Sample preparation for ^•^NO_2_ measurements

Rat pancreata were homogenized in 200 mM ice cold phosphate buffer, pH 7.4. The cytosolic fraction was obtained after 1 hr of centrifugation at 100.000 x g and used for NO_2_ assessment.

#### Samples for microscopic examination

The excised pancreata were immediately cooled on ice, cleaned from fat and lymphatic nodes, and weighed. Pancreatic tissue fragments measuring about 1 mm in the greatest dimension were incubated with 2.5% glutaraldehyde dissolved in 0.1 M sodium cacodylate buffer, pH 7.4, for 2 hrs. Specimens were rinsed extensively in the buffer and post-fixed by incubation with 2% OsO_4_. After fixation the samples were dehydrated in a graded series of alcohols and then were embedded in Epon 812. Ultrathin sections were stained with 5% uranyl acetate and lead citrate and examined using a JEM 1200 EX II electron microscope. Electron microscopic slides were examined by a pathologist.

### 3.3. Spectroscopic Measurements

Electronic absorption spectra were recorded using a Perkin-Elmer Lambda 18 Instrument with a scan accuracy of 1 nm, a 1nm slit width, and a scanning rate of 120.00 nm min^−1^. Kinetic measurements were carried out using an Applied Photophysics SX-17MV spectrophotometer using a stopped-flow technique.

### 3.4. Determination of Nitric Dioxide Concentration

A solution of the complex used to determine NO_2_ concentration was prepared by mixing 0.5 mL of *cis*-[Cr(C_2_O_4_)(AaraNH_2_)(OH_2_)_2_]^+^ (c = 10^−3^ M) with 2 mL of 0.2 M MES and 2 mL of 2 M NaClO_4_. The temperature of the solution was maintained at 20 °C with an accuracy of ±0.1 °C. Nitric dioxide concentrations were computed using Origin 6.0 software on the basis of absorbance variations at 541 nm using the non-linear least squares method [8].

### 3.5. Biochemical Assays

Protein concentration in the blood serum and cytosolic fraction was determined by the biuret method [20]. Serum amylase activity was measured according to the method of Caraway [21].

### 3.6. GSH Assessment

GSH levels in the cytosol of pancreatic acinar cells were quantified as described by Baker *et al.* [22]. In short, GSH was oxidized with DTNB (5′,5′-dithiobis(2-nitrobenzoic acid)) according to the equation: 2 GSH + DTNB → GSSG + 2 TNB. Formation of TNB was measured at 405 nm using a spectrophotometric microplate reader.

### 3.7. Statistical Analysis

Results are expressed as mean ± standard deviation (SD). Student’s *t* unpaired test was used to determine the statistical significance between compared experimental groups. *p* values are presented with obtained data.

## 4. Conclusions

Our development of new molecular biosensors selectively assessing cellular NO_2_ levels creates new possibilities for NO_2_ use as a diagnostic biomarker as well as a therapeutic target for future intervention. The prevention of GSH depletion in acinar cells during inflammatory processes is of potential clinical relevance. Despite the relatively well defined histological progression of AP in animal models, the intracellular events leading to cellular apoptosis and necrosis remain inadequately defined. Several studies suggested that oxidative stress may be an important trigger in the pathogenesis of AP. Glutathione, an essential component of defense against oxidative stress, seems to be a target of NO_2_ oxidative chemistry. Our findings suggest that NO_2_ directly, and not as a consequence of pancreatic injury during AP, induces glutathione depletion. Additionally, the nitrogen dioxide scavenger 4-OH-TEMPO seems to effectively prevent or markedly alleviate ultrastructural and biochemical changes observed in l-arginine induced AP in rat.
